# Use of Multiparametric and Biparametric Magnetic Resonance Imaging in Bladder Cancer Staging: Prospective Observational Study and Analysis of Radiologist Learning Curve

**DOI:** 10.3390/jcm13226955

**Published:** 2024-11-18

**Authors:** F. Piramide, M. Sica, G. Fondello, G. Mesterca, L. Ferrando, N. Ziani, M. Ortenzi, G. Grosso, B. Desana, P. Frattoni, S. De Cillis, A. Piana, D. Amparore, E. Checcucci, C. Fiori, S. Cirillo, F. Porpiglia, M. Manfredi

**Affiliations:** 1 Department of Oncology, Division of Urology, University of Turin, San Luigi Gonzaga Hospital, 10043 Turin, Italy; 2 Department of Surgery, Division of Urology, IRCCS Candiolo Cancer Institute, 10060 Turin, Italy; 3 Department of Radiology, Mauriziano Hospital, 10128 Turin, Italy

**Keywords:** bladder cancer, magnetic resonance imaging, VI-RADS score, learning curve

## Abstract

**Background**: Nowadays, thanks to the introduction of the VI-RADS scoring system, mpMRI has shown promising results in pre-TURBT assessment of muscular invasiveness of BCa, even if its application in everyday practice is still limited. This might be due to a lack in the literature about the learning curve of radiologists and about the characteristics of the exam. With the aim to reduce scan time and patient discomfort while maintaining diagnostic accuracy, bpMRI has been introduced as a possible alternative to mpMRI in this group of patients. This study reports a single-center experience using mpMRI and the VI-RADS scoring system to differentiate NMIBC from MIBC. The primary aim of the study is to assess diagnostic accuracy of mpMRI using the VI-RADS scoring system. The secondary aim is to evaluate the learning curve of an experienced mpMRI radiologist. Additionally, we perform a retrospective assessment of the same group of patients evaluating only DWIs and T2-weighted images, as they underwent bpMRI, to compare the performance of mpMRI and bpMRI. **Materials and Methods:** From 11/2021 to 11/2023, patients with suspected newly diagnosed BCa were enrolled in this prospective study. All patients underwent mpMRI prior to TURBT in a highly specialized radiology center for MRI. According to VI-RADS, a cutoff of ≥3 was assumed to define MIBC. Histological TURBT reports were compared with preoperative VI-RADS scores to assess the accuracy of mpMRI in discriminating between NMIBC and MIBC. Furthermore, to assess the learning curve of the reading radiologist we analyzed the rate of patients correctly classified as MIBC at MRI. Finally, we evaluated the performance of a hypothetic biparametric MRI in classifying our cohort according to VI-RADS score and compared it with mpMRI performance by using DeLong’s test. Data analysis was performed using Jamovi software v.2.3 and R software v.4.2.1. **Results:** A total of 133 patients were enrolled. mpMRI showed sensitivity and specificity of 86% (95% confidence interval [CI]: 64–97) and 95% (95% CI: 89–98), respectively. The learning curve analysis of the reading radiologist showed that the rate of patients correctly classified as MIBC rapidly increases reaching its plateau after 40 cases. The hypothetic bpMRI showed a sensitivity of 76% (95% CI: 53–92) and a specificity of 93% (95% CI: 86–97), with no significant difference with mpMRI performance (*p* = 0.10). **Conclusions:** Our study confirms the effectiveness of MRI, particularly with the VI-RADS scoring system, in differentiating NMIBC from MIBC. The learning curve analysis underscores the importance of radiologist training in optimizing diagnostic accuracy. Future research should focus on enhancing the sensitivity of bpMRI and further validating these findings in larger and multicentric studies.

## 1. Introduction

The most frequent presentation of bladder cancer (BCa) at diagnosis is represented by non-muscle invasive disease (NMIBC), categorized as Ta-T1 according to the TNM (tumor, node, and metastasis) classification system [1,2,3]. However, this group of patients shows significant heterogeneity, with different recurrence (up to 80%) and progression (up to 50%) rates and a non-negligible cancer mortality rate ranging from 16–23% at 5 years after conservative treatment based on risk profiles [2,4,5,6,7].

On the other hand, patients diagnosed with muscle-invasive bladder cancer (MIBC) face a substantial risk of metastatic progression. The 5-year overall survival rate after radical cystectomy (RC) for patients with BCa confined to the organ (pT2) stands at 68%, in contrast to 25–30% for those with extravesical extension [3,8].

Therefore, it is pivotal to properly classify these patients at diagnosis in order to offer them the correct treatment pathway and maximize their survival chances [9,10]. In this scenario, initial transurethral resection of bladder tumor (TURBT) has a central role, representing the diagnostic tool for both NMIBC and MIBC and also the first therapeutic step in the case of NMIBC.

Following the purpose to correctly classify patients harboring BCa, European Association of Urology guidelines advocate for a second look and resection (re-TURBT) 2–6 weeks following the primary TURBT for all T1 tumors and instances where detrusor muscle was not included in the sample [2]. This is crucial as approximately 7–30% of NMIBC cases are understaged post-TURBT, a figure that escalates to 45% when detrusor muscle is omitted from the resection [2].

Multiparametric magnetic resonance imaging (mpMRI) for BCa is evolving, offering increasingly accurate high-resolution tissue contrast capable of finely distinguishing bladder wall layers [11]. Recent experiences, both retrospective and prospective, have demonstrated promising outcomes with the VI-RADS (Vesical Imaging Reporting and Data System) scoring system, proving its reliability as an imaging-guided approach to assess muscular invasiveness pre-TURBT [12,13].

This system enhances diagnostic accuracy, reduces variability in MRI interpretation among radiologists, and improves communication within multidisciplinary teams. Furthermore, by identifying patients with high-risk MIBC preoperatively, VI-RADS scoring can guide treatment planning, potentially avoiding unnecessary procedures and reducing patient morbidity.

In clinical practice, however, the adoption of VI-RADS is often limited by the learning curve associated with its application. This is particularly relevant for radiologists who may be experienced with general mpMRI but less familiar with bladder cancer-specific imaging nuances [14]. However, only few data were reported [15] concerning the learning curve of radiologists for bladder MRI interpretation, leaving this question partially unsolved.

Besides the standard mpMRI, in recent years biparametric MRI (bpMRI) has emerged as an alternative option, which, focusing on fewer sequences (T2-weighted imaging [T2WI] and diffusion-weighted imaging [DWI]), is able to reduce scan time and patient discomfort while maintaining diagnostic accuracy [16].

This study aims to report our single-center experience using mpMRI and the VI-RADS scoring system in differentiating NMIBC from MIBC within a prospective cohort of patients with suspected BCa, providing a real-life overview of VI-RADS score performance. The secondary aim was to evaluate the learning curve of an experienced MRI radiologist in correctly classifying bladder tumors using the VI-RADS score, trying to fix the existent lack in current literature.

Finally, we retrospectively evaluate the diagnostic accuracy of a hypothetical bpMRI in correctly classifying this cohort of patients and to compare its performance with mpMRI.

## 2. Patients and Methods

### 2.1. Patient Population

From November 2021 to November 2023, consecutive patients with suspected newly diagnosed BCa scheduled for TURBT at our center were identified and enrolled in this prospective study. This study received formal Institutional Review Board and Ethical Committee approval (Ethical Committee name: San Luigi Gonzaga Hospital; approval code: 133/2021; approval date: 20 May 2021). The study was conducted in line with the European Urology and Good Clinical Practice guidelines according to ethical principles laid down by the latest version of the Declaration of Helsinki. All patients enrolled in this study provided informed written consent.

Exclusion criteria included previous history of BCa and contraindication to perform mpMRI (i.e., patients with impaired renal function; patients with claustrophobic disorder; patients with MR “unsafe” or “conditional” devices; and patients who could not attain adequate bladder distension).

All patients deemed eligible underwent mpMRI of the bladder and suspected lesions were evaluated by one experienced radiologist following the VI-RADS scoring system.

After the first TURBT, patients with high-risk Ta-T1 NMIBC (according to EAU guidelines) underwent re-TURBT and diagnostic accuracy of the VI-RADS score was evaluated again in this subgroup.

All demographic, radiological, and pathological variables were collected and analyzed.

### 2.2. Imaging Acquisition Protocol

All patients underwent the same mpMRI protocol using a 3 Tesla scanner (Philips, Ingenia, Amsterdam, The Netherlands), while a small proportion of examinations (7.3%) was acquired with a 1.5 Tesla scan (Philips, Ingenia) due to incompatibilities with medical devices (e.g., pacemakers) at 3 Tesla magnetic field, without however leading to significant changes in image quality or interpretation of results.

According to VI-RADS, the acquisition protocol included morphological T2-weighted sequences on three planes (axial, coronal, and sagittal), diffusion-weighted imaging acquired in the axial plane with multiple b-values (b = 0–800–1000), used to create the apparent diffusion coefficient (ADC) map, and dynamic contrast-enhanced images with fat-sat sequences (3D T1 gradient echo), before and after gadolinium-based contrast medium injection, acquired in the axial plane with a temporal resolution of 6 s (see Supplementary Table S1 for parameter settings).

An antispasmodic agent i.m. was administered to reduce bladder wall motion artifacts, and all the patients were asked to drink 500–1000 mL of water 30 min before the exam in order to obtain adequate bladder distension.

All exams were reviewed by an MRI radiologist with more than 15 years of expertise in urogenital and body imaging although with a limited experience in bladder MRI and the VI-RADS reporting system.

A VI-RADS cutoff score of ≥3 was assumed to define MIBC, and the final suspicion of muscular invasion was decided by DWI and DCE, using T2 imaging for morphological evaluation, given the high spatial resolution in assessing the integrity of muscularis propria.

In the case of discordance between T2 and DCE sequences with deviation greater than or equal to two categories, DWI improved accuracy as long as the image quality of the sequences was optimal [12].

### 2.3. Statistical Analysis

Continuous variables are summarized as median and interquartile range [IQR]. Categorical variables are reported as frequencies (percentages).

In order to evaluate the efficacy of mpMRI in distinguishing between NMIBC and MIBC (aim I), we computed sensitivity, specificity, positive predictive value (PPV), and negative predictive value (NPV) for the entire patient cohort who underwent mpMRI prior to TURBT. TURBT results were considered as the standard of reference for LR-NMIBC, re-TURBT for HR-NMIBC, and RC for MIBC. The performance of mpMRI was analyzed using receiver operating characteristic (ROC) curve analysis.

To assess the learning curve of the reading radiologist, we analyzed the rate of patients correctly classified as MIBC at MRI (such as VI-RADS score ≥ 3) and the rate of upstaging (patients with VI-RADS score < 3 resulted as MIBC at TURBT) and downstaging (patients with VI-RADS score ≥ 3 classified as NMIBC at TURBT) after TURBT. The experience of the radiologist was coded as the number of bladder MRIs read by the radiologist before the index patient’s MRI. Radiologist experience was entered as a continuous variable, using restricted cubic splines with knots at the tertiles to create a non-linear relationship between experience and the outcomes of interest mentioned above [17].

Finally, to assess the performance of a hypothetic bpMRI in classifying our cohort of patients according to the VI-RADS score (such as using only T2 and DWI images-based information), the reading radiologist reviewed all the studies blinded to the previous reports. To provide enough memory extinction, this retrospective review was conducted 6 months after the end of the enrollment of patients.

Data analysis was performed using Jamovi software v.2.3 and R software v.4.2.1.

## 3. Results

### 3.1. Patient Characteristics

A total of 140 consecutive patients suspected of having bladder neoplasms underwent MRI scans at a single center. Out of these patients, five (3.5%) were excluded from the final analysis due to undergoing TURBT before MRI, while two (1.75%) were excluded because histology revealed an inverted papilloma. Hence, the final sample comprised 133 patients. The median age was 71 years (interquartile range, 64–77 years), with 111 (83.5%) males and 22 (16.5%) females.

The percentage of smokers was 81.1% in patients with NMIBC and 81.3% in patients with MIBC while patients with CCI ≥ 5 comprised 46 (39.3%) and three (18.7%) for NMIBC and MIBC, respectively. Patients who had reported macrohematuria comprised 66 (56.4%) in the NMIBC category and 10 (62.6%) in the MIBC category. Table 1 provides a summary of the patient demographics.

The VI-RADS-related pathological findings are summarized in Table 2. Histologic examination in patients who had VI-RADS 1 always revealed NMIBC disease while in VI-RADS 5 100% of patients had MIBC disease with perfect MR-pathologic data concordance. In VI-RADS 2, only 7.0% (five patients) had upgrading disease on histological examination. In patients with VI-RADS 4 and VI-RADS 5, the detrusor was always represented in the specimens analyzed.

### 3.2. Differentiation Between NMIBC and MIBC at First and Re-TURBT

**Figure 1:**
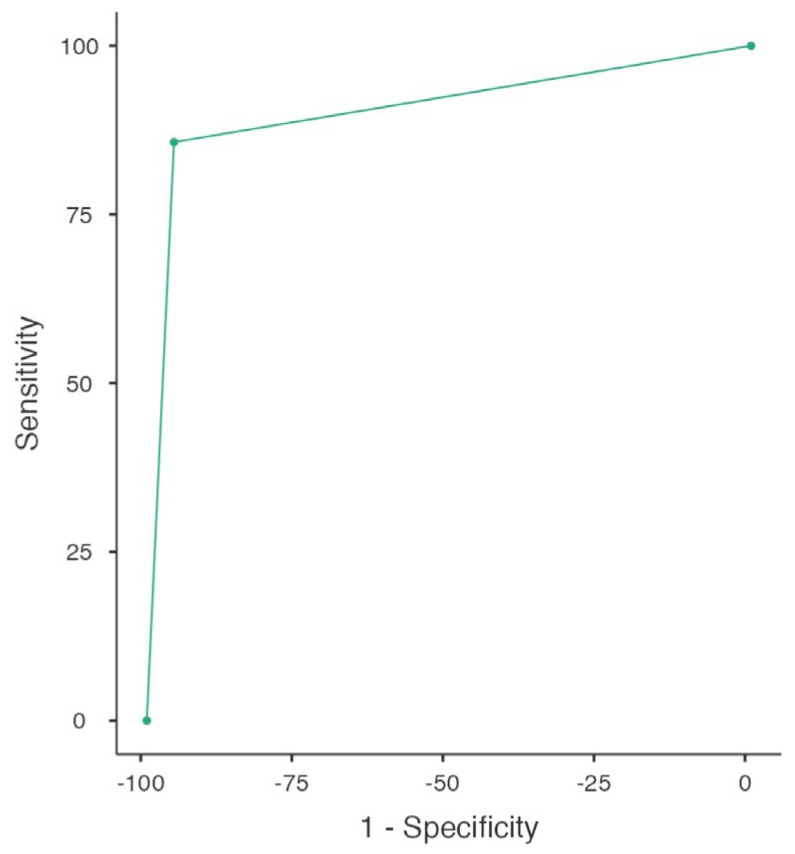
ROC curve representing VI-RADS score performance in discriminating NMIBC from MIBC at initial TURBT (whole cohort; *n* = 133).

The performance of MRI in differentiating NMBIC and MIBC at first TURBT was found to have a sensitivity of 86% (95% CI: 64–97) and a specificity of 95% (95% CI: 89–98). The PPV and NPV were 75% (95% CI: 65.8–86.7) and 97% (95% CI: 93.3–99.1), respectively, with an accuracy of 93%. In this setting the area under the curve (AUC) was 0.90. The ROC analysis is reported in Figure 1.

**Figure 2:**
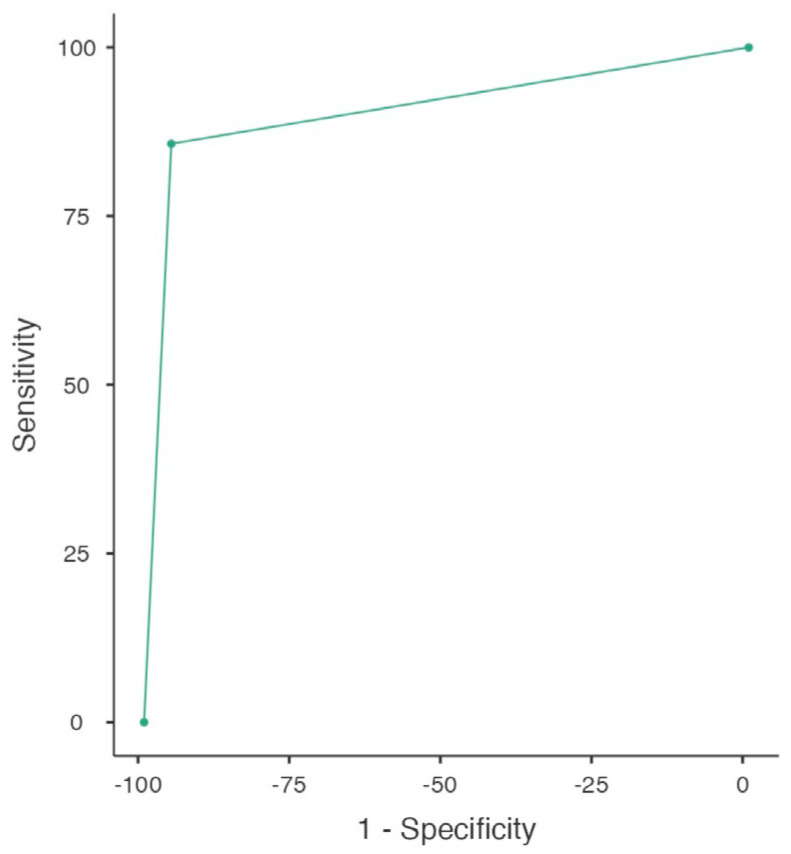
ROC curve representing VI-RADS score performance in discriminating NMIBC from MIBC at re-TURBT (whole cohort; *n* = 68).

Similar results were provided by the analysis of the subgroup of HR-NMIBC who underwent re-TURBT. In this cohort of patients (*n* = 68), the MRI reached a sensitivity of 80% (95% CI: 44–97) and a specificity of 95% (95% CI: 84–99). Furthermore, when a VI-RADS score ≥3 was diagnosed, an MIBC was effectively found at re-TURBT in 66.7% of patients (PPV, 95% CI: 52.4–90.1), whilst when no sign of muscle involvement was present, the re-TURBT was negative in 97.6% of cases (NPV, 95% CI: 90.5–99.3), reaching an accuracy of 94%. The AUC was 0.88 and the ROC analysis is reported in Figure 2.

### 3.3. Learning Curve Assessment

**Figure 3:**
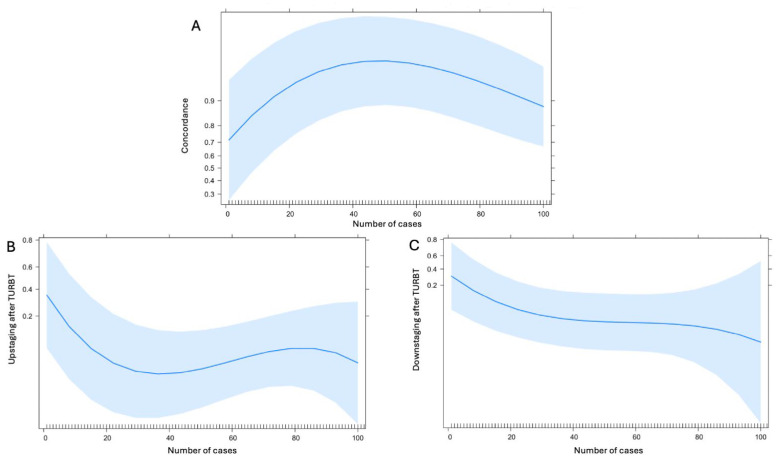
Radiologist learning curve analysis evaluating the concordance of MRI and TURBT (**A**), the upstaging rate (**B**), and the downstaging rate (**C**).

The learning curve analysis of the reading radiologist showed that the rate of patients correctly classified as MIBC at MRI (such as VI-RADS score ≥3) rapidly increases reaching its plateau after 40 cases (Figure 3A). Focusing on the rate of upstaging (patients with VI-RADS score <3 resulted as MIBC at TURBT) after TURBT, the learning curve analysis showed an early drop after 20–30 cases (Figure 3B) whilst the downstaging rate after TURBT showed to be flatter but with a constant decrease over time (Figure 3C).

### 3.4. Biparametric MRI Performance Differentiation Between NMIBC and MIBC at First and Re-TURBT

Overall, the use of T2- and diffusion-weighted images only (bpMRI) to score bladder lesions according to the VI-RADS system showed a sensitivity of 76% (95% CI: 53–92) and a specificity of 93% (95% CI: 86–97). The PPV and NPV were 66.7% (95% CI: 47–82) and 95.4% (95% CI: 87–98), respectively, with an accuracy of 90%. In this setting, the AUC was 0.85. DeLong’s test showed no significant difference between mpMRI and bpMRI performance (*p* = 0.10).

In HR-NMIBC subgroup bpMRI confirmed a good sensitivity of 67% (95% CI: 15–95) and a specificity of 95% (95% CI: 81–99). The PPV and NPV were 60% (95% CI: 35–89) and 95% (95% CI: 91–99), respectively, with an accuracy of 89% and an AUC of 0.78. Again, DeLong’s test showed no difference between the two methodologies (*p* = 0.32).

## 4. Discussion

In this study, we evaluated the effectiveness of MRI in differentiating non-muscle-invasive bladder cancer (NMIBC) from muscle-invasive bladder cancer (MIBC) in a cohort of 133 consecutive patients harboring BCa.

Our study found that MRI had a sensitivity of 86% and a specificity of 95% in distinguishing NMIBC from MIBC at the first TURBT, with a positive predictive value (PPV) and negative predictive value (NPV) of 75% and 97%, respectively. The overall accuracy was 93%, and the area under the curve (AUC) was 0.90. These results are consistent with recent literature, which reports sensitivity ranging from 60% to 85% and specificity from 80% to 95% for MRI in diagnosing MIBC [20,21,22,23]. Our specificity of 95% and high accuracy of 93% underscore MRI’s reliability in ruling out muscle invasion when it is not present, similar or slightly higher to the results reported in literature by VI-RADS referral centers [13,24], who found a specificity of 86–93% and an accuracy of 91%.

For the subgroup of patients with high-risk NMIBC undergoing re-TURBT, MRI sensitivity was slightly lower (80%), but specificity remained high (95%). This aligns with other studies [20,22] where authors reported a sensitivity of 65% and a specificity of 92% in similar populations. Our accuracy of 94% and an AUC of 0.88 suggest that MRI is useful also in this clinical setting although requiring rigorous follow-up.

The learning curve analysis for the radiologist interpreting MRI scans according to the VI-RADS scoring system revealed significant improvements over time. The rate of correctly classifying patients as having MIBC (VI-RADS score ≥ 3) rapidly increased, reaching a plateau after approximately 40 cases.

In current literature there is a lack of studies analyzing the radiologists’ learning curve in VI-RADS score assessment, and only one study, focused on residents’ learning curve, was available, to the best of our knowledge. Here, the authors showed that the diagnostic accuracy of residents increased significantly after interpreting around 100–150 cases [15]. The need for a different caseload could be read as an indirect confirmation that 40–50 cases might be enough for an experienced radiologist, with a strong background in pelvic MRI, for a good handling of the VI-RADS scoring system.

Furthermore, our learning curve analysis for upstaging and downstaging rates post-TURBT showed a rapid reduction in upstaging rates after 20–30 cases, while downstaging rates decreased more gradually over time. This highlights the importance of radiologist experience and training in minimizing diagnostic errors and improving patient outcomes.

Focusing on the use of biparametric MRI (bpMRI), which includes only T2- and diffusion-weighted images, showed a sensitivity of 76% and a specificity of 93% for detecting MIBC according to the VI-RADS scoring system, indicating that it remains a viable option while reducing scan time and patient discomfort. Notably, DeLong’s test showed no significant difference between the diagnostic performance of mpMRI and bpMRI, suggesting that bpMRI could be a practical alternative without compromising accuracy. Overall, these findings advocate for the integration of MRI, particularly bpMRI, into clinical protocols for initial bladder cancer staging and follow-up assessments.

Our study is not free of limitations. First of all, the final analysis included 133 patients, which, although adequate, might not capture the full spectrum of bladder cancer variability, especially concerning the variant histology of BCa, already proved to have important impact on prognosis and to be a challenge for radiologists [16,25,26]. Secondly, the study was conducted at a single center, which may limit the generalizability of the findings to other settings and populations. Another limit could be that this study focused on the learning curve of an experienced radiologist, which may not be applicable to less experienced practitioners or generalize to all radiologists. Finally, we acknowledge that our study’s assessment of bpMRI was conducted retrospectively, although blinded to previous reports and after 6 months of memory extinction, which may limit the accuracy of our findings compared to a prospective analysis. Since bpMRI performance was evaluated retrospectively and without direct comparison during real-time clinical decision-making, the diagnostic performance might differ in practice.

On the other hand, our study provides a prospective cohort analysis, enhancing the reliability of our findings and reducing the risk of selection bias. Furthermore, our report is one of the first reported in the literature focusing on the VI-RADS learning curve of an experienced MRI radiologist. This data could be helpful for pushing further the widespread use of the VI-RADS scoring system, encouraging other radiologists in adopting this tool. Finally, our enrolling time was relatively shorter in comparison with other series [22,27] where the study period was around 7 years and fewer patients enrolled.

Future studies should focus on investigating the impact of accurate MRI staging on long-term clinical outcomes and survival rates, which would provide a more comprehensive understanding of its benefits. Furthermore, a prospective design incorporating bpMRI directly into the diagnostic workflow could better evaluate its effectiveness, allowing for a more accurate comparison with mpMRI in real clinical settings. Adopting bpMRI in routine clinical practice could offer significant advantages, including reduced scan times and enhanced patient comfort, potentially leading to higher patient compliance. Shorter scan times can also improve patient throughput, allowing for a greater number of scans in high-demand radiology departments. Additionally, bpMRI might reduce costs associated with MRI by eliminating the need for gadolinium-based contrast agents, which are both costly and associated with specific patient contraindications. Meanwhile, we believe that the developing of standardized training programs for radiologists based on the learning curve analysis can further improve the diagnostic accuracy of bladder MRI.

## 5. Conclusions

Our study confirms the effectiveness of mpMRI, particularly with the VI-RADS scoring system, in accurately differentiating NMIBC from MIBC. The high specificity and accuracy reinforce VI-RADS as a reliable non-invasive diagnostic tool that can enhance preoperative staging and guide appropriate treatment planning. The learning curve analysis underscores the importance of targeted radiologist training, showing that proficiency in VI-RADS scoring can be achieved relatively quickly, particularly among experienced radiologists. These findings suggest that standardized training programs incorporating case-based learning could accelerate diagnostic accuracy and consistency, potentially making VI-RADS more accessible in routine practice.

Looking forward, future research should aim to validate these findings in larger, multicenter studies to enhance the generalizability of VI-RADS across various clinical settings. Additionally, prospective studies evaluating bpMRI as a feasible alternative to mpMRI could offer valuable insights into improving patient throughput and reducing diagnostic costs, potentially reshaping bladder cancer staging protocols.

## Figures and Tables

**Table 1:** Patients’ demographics. NMIBC: non muscular invasive bladder cancer, MIBC muscular invasive bladder cancer, TURBT trans-urethral resection of bladder tumor, WHO World Health Organization.

	NMIBC, *n* (%)	MIBC, *n* (%)
No. of patients	117 (88.0)	16 (12.0)
Age (mean)		
<70	52 (44.4)	5 (31.3)
≥70	65 (55.6)	11 (68.7)
Sex		
Male	105 (89.7)	6 (37.4)
Female	12 (10.3)	10 (62.6)
Smoking history		
No	18 (15.4)	2 (12.5)
Yes	95 (81.1)	13 (81.3)
Ex smoker	4 (3.5)	1 (6.2)
Charlson comorbidity index		
<5	71 (60.7)	13 (81.3)
≥5	46 (39.3)	3 (18.7)
Body mass index (Kg/m^2^)		
<18	3 (2.5)	1 (6.2)
18–25	43 (36.8)	6 (37.4)
>25	71 (60.7)	9 (56.4)
Hematuria		
No	25 (21.4)	3 (18.7)
Micro	26 (22.2)	3 (18.7)
Macro	66 (56.4)	10 (62.6)
WHO grading (2004) [18]		
Low Grade	51 (43.6)	1 (6.2)
High Grade	66 (56.4)	15 (93.8)
WHO grading (1973) [19]		
G1	9 (7.7)	0 (0)
G2	46 (39.3)	3 (18.7)
G3	62 (53.0)	13 (81.3)
No. of foci at TURBT		
1	62 (53.0)	
>1	55 (47.0)	

**Table 2:** Patients’ pathological features and VI-RADS assessment for bladder cancer (*n* = 133).

Variables, *n* (%) No. of Patients	VI-RADS 1 *n* = 36 (27)	VI-RADS 2 *n* = 71 (53.4)	VI-RADS 3 *n* = 11 (8.3)	VI-RADS 4 *n* = 8 (6.0)	VI-RADS 5 *n* = 7 (5.3)
Lesion diameter (cm)					
<3 cm	32 (88.9)	60 (84.5)	8 (72.7)	3 (37.5)	2 (40.0)
≥3 cm	4 (11.1)	11 (15.5)	3 (27.3)	5 (62.5)	5 (60.0)
TURBT pathologic stage					
Ta	25 (69.5)	25 (35.2)	2 (18.2)	0	0
T1	11 (30.5)	41 (57.8)	2 (18.2)	1 (12.5)	0
T2	0	5 (7.0)	7 (63.6)	7 (87.5)	7 (100)
TURBT pathologic grade					
Low grade	30 (83.3)	21 (29.8)	0	0	0
High grade	6 (16.7)	50 (70.2)	11 (100)	8 (100)	7 (100)
Detrusor muscle					
Present	31 (86.1)	60 (84.5)	7 (63.6)	8 (100)	7 (100)
Absent	5 (13.9)	11 (15.5)s	4 (36.4)	0	0

## Data Availability

The original contributions presented in the study are included in the article, further inquiries can be directed to the corresponding author.

## Supplementary Materials

The following supporting information can be downloaded at: https://www.mdpi.com/article/10.3390/jcm13226955/s1, Table S1: Parameter setting.
