# Supplementary material for: Use of Multiparametric and Biparametric Magnetic Resonance Imaging in Bladder Cancer Staging: Prospective Observational Study and Analysis of Radiologist Learning Curve
Source: J Clin Med. 2024 Nov 18;13(22):6955. doi: 10.3390/jcm13226955 (PMC11595213; doi:10.3390/jcm13226955)
Supplement: Supplementary file 1 [file jcm-13-06955-s001.zip › jcm-3187012-supplementary.pdf]

Supplementary Table S1. Parameter setting

	**T2**	**DWI**	**DCE**
**1.5 T**			
TE (ms)	120	85	1.97
TR (ms)	3200 - 5500	3749	4.1
Flip angle (degree)	90	90	10
FOV (cm)	23	25	30
Matrix	308 x 303	84 x 67	232 x 232
Slice thickness (mm)	3.5	3.5	3
Slice gap (mm)	0	0	0
Number of excitations	3	3	1
b values		0-800-1000	
**3 T**			
TE (ms)	80	80	1.95
TR (ms)	2500 - 5500	3000 - 5000	4.9
Flip angle (degree)	90	90	10
FOV (cm)	20	20	24
Matrix	400 x 399	80 x 79	200 x 200
Slice thickness (mm)	3	3	3
Slice gap (mm)	0	0	0
Number of excitations	1	5	1
b values		0-800-1000	
